# Circulating angiotensin peptides levels in Acute Respiratory Distress Syndrome correlate with clinical outcomes: A pilot study

**DOI:** 10.1371/journal.pone.0213096

**Published:** 2019-03-07

**Authors:** Raju Reddy, Isaac Asante, Siyu Liu, Pranay Parikh, Janice Liebler, Zea Borok, Kathleen Rodgers, Ahmet Baydur, Stan G. Louie

**Affiliations:** 1 Division of Pulmonary, Critical Care and Sleep Medicine, Keck School of Medicine, University of Southern California, Los Angeles, CA, United States of America; 2 Department of Clinical Pharmacy, School of Pharmacy, University of Southern California, Los Angeles, CA, United States of America; 3 Hastings Center for Pulmonary Research, Keck School of Medicine^3^, University of Southern California, Los Angeles, CA, United States of America; Escola Paulista de Medicina, BRAZIL

## Abstract

**Rationale:**

We propose renin angiotensin system (RAS) peptides are critical in wound reparative processes such as in acute respiratory distress syndrome (ARDS). Their role in predicting clinical outcomes in ARDS has been unexplored; thus, we used a targeted metabolomics approach to investigate them as potential predictors of outcomes.

**Methods:**

Thirty-nine ARDS patients were enrolled within 24 hours of ARDS diagnosis. Plasma RAS peptide levels were quantified at study entry and 24, 48 and 72 hours using a liquid chromatography-mass spectrometry based metabolomics assay. RAS peptide concentrations were compared between survivors and non-survivors, and were correlated with clinical and pulmonary measures.

**Measurements and main results:**

Angiotensin I (Ang-I or A(1–10)) levels were significantly higher in non-survivors at study entry and 72 hours. ARDS survival was associated with lower A(1–10) concentration (OR 0.36, 95% CI 0.18–0.72, p = 0.004) but higher A(1–9) concentration (OR 2.24, 95% CI 1.15–4.39, p = 0.018), a biologically active metabolite of A(1–10) and an agonist of angiotensin II receptor type 2. Survivors had significantly higher median A(1–9)/A(1–10) and A(1–7)/A(1–10) ratios than the non-survivors (p = 0.001). Increased A(1–9)/A(1–10) ratio suggests that angiotensin converting enzyme II (ACE2) activity is higher in patients who survived their ARDS insult while an increase in A(1–7)/A(1–10) ratio suggests that ACE activity is also higher in survivors.

**Conclusion:**

A(1–10) accumulation and reduced A(1–9) concentration in the non-survivor group suggest that ACE2 activities may be reduced in patients succumbing to ARDS. Plasma levels of both A(1–10) and A(1–9) and their ratio may serve as useful biomarkers for prognosis in ARDS patients.

## Introduction

Acute respiratory distress syndrome (ARDS) is characterized by disruption of the alveolar-capillary barrier leading to inflammation causing lung injury [1, 2]. Mortality rates range from 38.5–46.1 percent, with older age and disease severity being key risk factors for increased mortality [2, 3]. Given the high morbidity and mortality associated with ARDS, the development of biomarkers is important to identify patients at greatest risk for poor prognosis and outcome. Biomarkers such as plasma angiopoietin-2, Von-Willebrand factor, intracellular adhesion molecule 1 (ICAM-1), interleukin (IL-6), IL-8, protein C, and plasminogen activator inhibitor 1 (PAI-1) have been associated with clinically relevant outcomes [1–5]. However, these biomarkers may be limited by their specificity to particular disease conditions.

**Fig 1:**
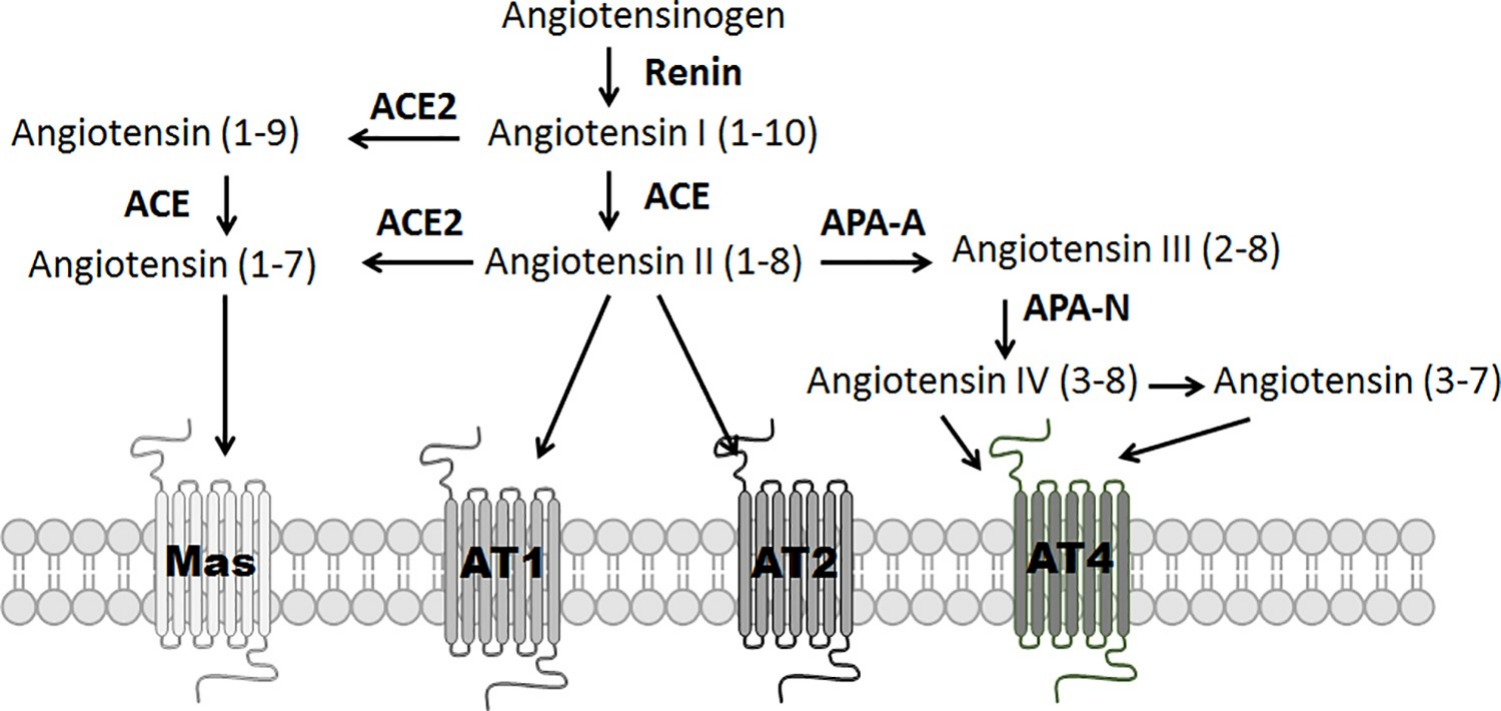
Metabolic pathway of angiotensinogen. This figure shows the major metabolites of angiotensinogen, the receptors they act on and the associated enzymes. AT4 is insulin regulated membrane aminopeptidase (IRAP).

The renin-angiotensin system (RAS) peptides have been a topic of interest for the last two decades related to their key role in respiratory conditions [6–8]. In the classical RAS pathway, angiotensinogen, the precursor of angiotensin I {A(1–10)}, is synthesized in the liver and converted to A(1–10) by renin (Fig 1). A(1–10) is further metabolized to angiotensin II {Ang II or A(1–8)}, a reaction mediated by angiotensin converting enzyme (ACE) which is found in lung endothelial cells. A(1–8) is an important regulator of hemodynamics, but has also been linked to tissue regeneration, remodeling, inflammation, and fibrosis [9]. In mouse models of ARDS, A(1–8) binding to Ang II receptor type 1a (AT_1a_) leads to impaired lung function and fibrosis, while treatment with an angiotensin receptor blocker (ARB) attenuates both inflammation and fibrosis [10]. In a human study, elevated circulating A(1–8) concentrations in influenza A (H7N9) pneumonia were associated with higher mortality rates [7].

In an alternative pathway, angiotensin converting enzyme II (ACE2) converts A(1–8) to angiotensin (1–7) {A(1–7)}, and A(1–10) is metabolized to angiotensin (1–9) {A(1–9)}, which is a ligand for Angiotensin II receptor type 2 (AT_2_) [11]. ACE2 is a cell membrane-associated enzyme expressed on lung endothelial and epithelial cells found in the heart and kidneys. Loss of ACE2 enzymatic activity leads to severe inflammation, impaired cardiac activity and renal injury [12]. Several studies showed that ACE2 deficiency led to A(1–8) accumulation while reducing A(1–7) production. A(1–7) promotes wound healing, regenerates tissues, and reduces reactive oxygen species (ROS) by binding to its cognate receptor, Mas [13]. In mouse models of ARDS, intravenous infusion of recombinant A(1–7) has been shown to attenuate the inflammatory response [8]. Similar to A(1–7), A(1–9) also has regenerative and anti-inflammatory properties through its binding to AT_2_ [14, 15]_._ A(1–10) is also a substrate of neprilysin (NEP) to form A(1–7). Thus, the balance among RAS peptide levels may be an important factor in determining outcomes following acute lung injury.

We have developed a quantitative metabolomics approach that allows us to specifically determine circulating levels of RAS peptides. The goal of this study was to apply this to determine whether changes in specific RAS peptides correlated with survival and whether these peptides and ratios between them could serve as biomarkers in predicting outcomes in patients with ARDS.

## Methods

The protocol was approved by the University of Southern California Institutional Review Board to evaluate RAS peptides in patients with a diagnosis of ARDS (HSC-16-00). The Berlin definition of ARDS was used [16]. After the patient or their designee had signed an informed consent, the patient’s history, demographic and clinical information were recorded and blood samples were collected. Data collected included PaO_2_/FiO_2_ (P/F) ratios, sequential organ failure assessment (SOFA) score, length of hospital stay, body mass index (BMI), and serum creatinine and plasma lactate concentrations from days 1 through 4 following intensive care unit (ICU) admission. In addition, blood samples were collected at study entry and 24, 48 and 72 hours thereafter to measure RAS peptides.

### Inclusion criteria

Patients’ aged 18–90 years who met ARDS criteria were enrolled into the study within 24 hours of diagnosis. Patients with ARDS caused by both pulmonary and extra-pulmonary causes such as pancreatitis and non-pulmonary sepsis were included.

### Exclusion criteria

Pregnant patients of any age were excluded because possible effects of pregnancy on RAS peptides are unknown.

### Blood sample collection

About 1 mL of blood was collected with 10 μl of protease inhibitor cocktail comprised of 450 mg 1,10 phenanthroline, 15 mg pepstatin A, 37.5 mg enalapril maleate, 700 mg disodium ethylenediaminetetraacetate dihydrate, 17.4 mg phenylmethane sulfonyl fluoride and 1 mL of deionized water. Following collection, samples were gently placed on ice and centrifuged at 1000 rpm at 4°C for 15 minutes. The clarified plasma samples were transferred into cryovials and frozen at -80°C until analysis.

### Metabolomics assay

A validated method to quantify RAS peptides [A(1–12), A(1–10), A(1–9), A(1–8), A(1–7), A(2–8), A(3–8), and A(1–5)] in plasma samples was used. In brief, 40 μL of 1000 ng/mL NorLeu^3^-A(1–7) was added as the internal standard into 150 μL of plasma sample. Samples were then alkalinized by adding 700 μL 5% ammonium hydroxide solution and layered onto pre-conditioned solid phase extraction cartridges (Oasis 1cc MAX P/No. 186001883, Waters, MA, USA). The analytes were washed with water and methanol, and eluted using 1 mL methanol with 2% formic acid. The eluted samples were evaporated to dryness under a filtered steady stream of nitrogen gas. The residue was reconstituted using 50 μL of 0.2% formic acid and 45 μL of the reconstituted material was injected into a Shimadzu LC-20AD high pressure liquid chromatograph (HPLC) (Shimadzu, Japan) linked to an API 4000 mass spectrometer (AB Sciex, MA, USA) operating in the positive mode.

RAS peptide analytes were separated using a Hypersil Gold C18 column (P/No. 25005–052130, Thermo Scientific, USA) with 5 μm particle size and dimensions of 50 x 2.1 mm. Component A consisted of water with 0.5% formic acid, while component B was acetonitrile with 0.5% formic acid. A gradient program was used, where the concentration of component B was kept at 5% for 0.5 min initially and increased to 70% over 4.5 min. Component B was further increased to 90% over 2 min, and held at 90% for an additional 2 min and then returned to 5% for 2 min. The level of each analyte was determined using their unique multiple reaction-monitoring (MRM) transitions.

### Statistical analysis

Statistical analysis was performed using SAS 9.4 (SAS Institute, Cary NC). When appropriate, either Mann-Whitney rank sum test or Chi-square test was used. Baseline RAS peptides were compared between survivors and non-survivors. Plasma RAS levels were transformed to natural logarithms prior to regression analysis to render the data more normally distributed by visual inspection and Q-Q plots against a normal distribution. We then tested the association between ARDS survival as an outcome, and continuous and dichotomous variables (including RAS peptides), as predictors using binary logistic regression models [17]. Regression diagnostics were evaluated to ascertain the fit of the prediction model. Statistical significance was defined as p < 0.05. Serum creatinine was used to estimate glomerular filtration rate (eGFR) using the Modification of Diet in Renal Disease (MDRD) equations. Patients were grouped as either kidney failure or no kidney failure groups using a cut-off eGFR of <30 mL/min/1.73m^2^. Baseline RAS peptides were compared between the no renal failure and the renal failure groups.

## Results

### Demographics

This was a non-interventional observational study involving 39 patients that were consecutively enrolled over 20 months. Patient characteristics at study entry are summarized in Table 1. At follow up on 30 days after initial admission, there were 20 survivors (51%) and 19 non-survivors (49%). These results were consistent with clinical outcomes observed in other ARDS studies [2, 3]. Amongst the 20 survivors, 16 patients were discharged to home or to a rehabilitation facility. The four remaining patients survived their initial ICU admission, then developed another bout of pneumonia and succumbed to their illness. No follow-up RAS peptides were measured in these patients. At study entry, most enrolled patients had either moderate or severe ARDS as determined by P/F ratios (Table 1). Survivors had statistically lower serum lactate and SOFA scores when compared to those who succumbed to ARDS (P<0.05) at study entry, 24 and 48 hours. In addition, survivors were less likely to need vasopressor support to maintain blood pressure. There were no differences amongst P/F ratios, serum albumin or acute kidney injury between the two groups. The length of hospital stay was significantly longer in the survivor group compared to the non-survivor group (34 vs 10 days, p = 0.004).

**Table 1:** Anthropometric and physiologic data of ARDS patients at study entry.

Variable	Survivors (n = 20)	Non-survivors (n = 19)	p-value*
**Age, years**	51.5 (43.8–60.5)	55 (47.5–61.5)	0.71
**Gender (Male), n (%)**	9 (45)	16 (84)	0.011
**Body Mass Index (BMI), Kg/m**^**2**^	24.7 (28.1–30.9)	25.6 (22.8–30.9)	0.38
**Length of Stay (LOS), days**	34.0 (16.5–57.0)	10.0 (8.0–19.0)	0.004
**Diagnoses**			
*Pulmonary ARDS*	14	14	
Extra-pulmonary ARDS**	6	5	
**Severity of ARDS**			
*Mild*, *n (%)*	3 (15)	2 (10)	
*Moderate*, *n (%)*	8 (40)	10 (53)	
*Severe*, *n (%)*	9 (45)	7 (37)	
**Serum Lactate, (mmol/L)**	1.5 (1.1–2.4)	3.8 (1.6–9.0)	0.013
**PaO**_**2**_**/FiO**_**2**_ **(P/F) ratio**	117.8 (83.5–158.0)	128.0 (87.5–166.7)	0.703
**Mean arterial pressure, mmHg**	71 (60–90)	65 (55–75)	0.004
**Acute Kidney Injury, n (%)**	8 (40)	12 (63)	0.156
**Renal Replacement Therapy, n (%)**	4 (20)	8 (42)	0.142
**Sequential Organ Failure Assessment (SOFA) Score**	9 (8–12)	12 (11–13)	0.007
**Vasopressors/Inotropes, n (%)**	8 (40)	18 (95)	<0.001

Values are reported as median values with interquartile ranges. Variables with statistically significant differences between survivor and non-survivor groups include gender, serum lactate, and SOFA score at study entry.

*P values were determined using Mann Whitney U test for continuous variables and chi-square test for dichotomous variables.

**Extra-pulmonary ARDS is defined as ARDS due to sepsis from a non-pulmonary source such from a urinary source or pancreatitis.

### Levels of RAS peptides

Plasma samples were collected at study entry and every 24 hours for a total of four blood collections. Circulating concentrations of RAS peptides at study entry are summarized in Table 2 and Table 3. A(1–10) levels were significantly higher in the non-surviving group at study entry and at 24, 48 and 72 hours (p<0.05). No significant differences in A(1–9), A(1–8) and A(1–7) were noted between the two groups at any of the time points of blood sampling. In addition, there were no differences in levels of A(1–12), a surrogate marker of angiotensinogen, between the two groups. This suggests that higher levels of A(1–10) amongst non-survivors was likely due to decreased conversion of A(1–10) to further downstream products or increased biosynthesis. Increase synthesis is unlikely since A(1–12), a precursor for A(1–10) concentration was no different between the two outcomes.

**Table 2:** Median RAS peptide concentrations in ARDS patients at study entry.

RAS peptide	Median Concentration (ng/mL) (interquartile range)		
	Non-survivors (N = 19)	Survivors (n = 20)	p-value
**A(1–12)**	0.05 (0.05–0.64)	0.05 (0.05–1.97)	0.875
**A(1–10)**	5.86 (1.99–16.95)	2.33 (0.73–5.66)	0.012
**A(1–9)**	1.14 (0.10–2.82)	1.55 (0.55–3.08)	0.216
**A(1–8)**	0.38 (0.12–0.63)	0.76 (0.07–2.22)	0.298
**A(1–7)**	0.19 (0.12–0.61)	0.63 (0.08–1.07)	0.203

**Table 3:** Median RAS peptide concentrations in ARDS survivors (S) and non-survivors (NS) at 24, 48 and 72 hours.

RAS Peptide	Median Concentration (ng/mL)					
	24 hours		48 hours		72 hours	
	NS	S	NS	S	NS	S
**A(1–12)**	0.05	0.18	0.57	0.06	1.04	0.05
A(1–10)*	5.12	1.43	12.15	1.92	4.64	1.24
**A(1–9)**	0.89	0.98	0.97	1.54	3.46	1.42
**A(1–8)**	0.31	0.68	0.59	0.78	0.77	0.61
**A(1–7)**	0.21	1.08	0.83	0.97	0.81	0.88

* Only A(1–10) was significantly different with p-values of 0.015, 0.002 and 0.047 at 24h, 48h and 72h respectively.

When circulating concentrations of RAS peptides were compared based on kidney function, a significantly lower A(1–5) and A(1–7)/A(1–5) ratios were observed in patients with renal failure (p = 0.003 and p = 0.003 respectively, Table 4).

**Table 4:** Median RAS peptide concentrations and ratios between ARDS patients grouped by their renal failure status at study entry.

RAS peptide/ Ratio	Median concentration or ratio (interquartile range)		
	No Renal Failure (N = 17)	Renal Failure (n = 22)	p-value
**A(1–5)**	0.05 (0.05–0.14)	0.44 (0.09–0.73)	0.003
**A(1–9)**	1.88 (0.63–3.50)	0.69 (0.05–1.83)	0.025
**A(1–5)/ A(1–7)**	0.18 (0.12–1.25)	1.00 (0.46–3.11)	0.02
**A(1–9)/ A(1–10)**	0.64 (0.14–2.06)	0.10 (0.05–0.52)	0.023

### RAS peptide ratios (product/reactant)

A surrogate for ACE and ACE2 enzymatic activity can be computed using peptide ratios of product over reactant, thus providing an indirect measure of ACE and ACE2 enzymatic activities between survivors and non-survivors. As shown in Table 5, higher peptide ratios of A(1–9)/A(1–10) and A(1–8)/A(1–10) were also detected amongst survivors when compared to non-survivors (p<0.001). Although non-significant, we also found higher peptide ratios of A(1–7)/A(1–9) and A(1–7)/A(1–8) in survivors when compared to non-survivors, thus suggesting higher ACE and ACE2 enzymatic activity for survivors. These ratios may not have reached significance due to small sample size and high patient to patient variability. Lastly, we found higher ratios of A(1–7)/A(1–10) in the survivor group, suggesting a possibility of higher NEP activity amongst survivors. When renal function was used as a co-variate, a significantly higher A(1–10)/A(1–9) ratio (a marker for ACE2 activity) in the group with normal renal function as compared to patients with renal failure (p = 0.023, Table 4).

**Table 5:** Comparison of the ratios of circulating RAS peptides in ARDS at study entry.

*Ratio: Precursor→Product	Median Ratio (interquartile Range)		
	Non-survivors	Survivors	P-value
**A(1–12)→A(1–10)**	60.0 (2.05–315.99)	4.05 (0.89–29.93)	<0.001
**A(1–10)→A(1–9)**	0.08 (0.04–0.27)	1.00 (0.51–2.05)	<0.001
**A(1–10)→A(1–8)**	0.06 (0.01-.11)	0.74 (0.39–1.09)	<0.001
**A(1–9)→A(1–7)**	0.27 (0.04–1.09)	0.4 (0.09–0.64)	0.692
**A(1–10)→A(1–7)**	0.03 (0.01–0.08)	0.48 (0.14–0.97)	<0.001
**A(1–8)→A(1–7)**	0.63 (0.24–1.82)	0.85 (0.46–1.07)	0.655
**A(1–8)→A(2–8)**	5.8 (0.77–16)	1.54 (0.55–4.93)	0.093
**A(1–7)→A(1–5)**	1.00 (0.27–2.6)	0.29 (0.08–2.44)	0.070
**A(1–7)→aA(1–7)**	1.18 (0.37–9.12)	2.12 (0.91–4.85)	0.206
**A(2–8)→A(3–8)**	0.26 (0.10–0.93)	0.63 (0.14–1.00)	0.438

These ratios of product to precursor provide insights into the metabolic levels of the catalyzing enzymes. Ratios significantly different between survivor and non-survivor groups were A(1–12) **→**A(1–10), A(1–10) **→**A(1–9), A(1–10) **→**A(1–8) and A(1–10) **→** A(1–7) (p <0.001)

*Ratio is calculated as product concentration/precursor concentration.

### Association of peptide levels with clinical outcomes

No association was seen between plasma concentrations of A(1–12), A(1–10), A(1–9), A(1–8) and A(1–7) and P/F ratios at all blood collection time points from study entry to 72 hours. However, there was a significant association between A(1–7)/A(1–10) ratio and P/F ratio (p = 0.001). A significant association was also observed between survival, as an outcome and levels of A(1–9) and A(1–10), as independent variables. At study entry, the odds of surviving ARDS increased three fold when plasma A(1–9) concentration doubled (OR 2.24, CI 1.15–4.39, p = 0.018). In contrast, the odds of surviving decreased two-fold when plasma A(1–10) concentration was doubled (OR 0.36, CI 0.18–0.72, p = 0.004). Using these levels of A(1–10) and A(1–9) at study entry in a binary logistic regression model, we are able to predict ARDS survival with 79.5% accuracy (Table 6). This model was reproducible when 70% of the random sample was used as a training dataset and the remaining 30% as a validation data set.

**Table 6:** Multivariate logistic regression model for association with ARDS survival using A(1–9) and A(1–10) in their natural logarithm transformed forms as variables.

Variable	Coefficient	Odds Ratio	95% C.I.	p-value
**Ln[A(1–9)]**	0.81	2.24	1.15–4.39	0.018
**Ln[A(1–10)]**	-1.21	0.36	0.18–0.72	0.004

## Discussion

In this cohort, 19/39 (48.7%) ARDS patients succumbed to their disease similar to other published reports [2, 3]. In univariate analysis, a greater mortality risk was associated with higher SOFA scores, plasma lactate levels and elevated A(1–10) levels at study entry. However, in multivariate analysis, A(1–10) and A(1–9) levels at study entry showed an association with mortality. Specifically, higher A(1–10) levels were associated with increased mortality while higher A(1–9) levels were associated with decreased mortality. Also, higher A(1–9)/A(1–10) and A(1–7)/A(1–10) ratios were observed in the surviving group. A binary regression model using levels of A(1–10) and A(1–9) at study entry could accurately predict outcomes in ARDS with 79.5% confidence.

To date, no study has used the metabolomics approach to determine circulating levels of RAS peptides as predictors of survival outcomes in ARDS. Using this approach, we found that plasma A(1–10) levels were predictive for clinical outcome at study entry. Although a statistically significant difference was also achieved at 72 hours, we avoided possible bias arising from deaths in one of the groups by focusing on the predictive capacity using RAS peptides at study entry. No statistically significant differences were found in plasma levels of A(1–8) and A(1–7) between the two groups at any time point. However, upon analyzing peptide ratios (product/reactant), there were suggested differences in the two groups’ comparative ability to form the active RAS metabolites.

To determine whether higher levels of A(1–10) amongst non-survivors is a consequence of increased biosynthesis or reduced metabolism, we examined levels of both precursor peptides and downstream metabolic peptides. A(1–12) was used as a surrogate marker for angiotensinogen. There was no difference in A(1–12) between the two outcomes group, suggesting that higher levels of A(1–10) in the non-survivor group were not likely due to its increased synthesis. Furthermore, levels of downstream products such as A(1–9), A(1–8) and A(1–7) were lower in the non-survivor group, but not statistically different. Despite the lack of statistical significance, survivors had approximately three times higher median plasma A(1–7) levels suggesting the metabolism to form bioactive peptides may be impeded. Taken together, these findings suggest that non-survivors have reduced metabolism of A(1–10) which could be due to reduction in ACE and/or ACE2 enzymatic activities as a consequence of more severe lung endothelial and epithelial injuries in the non-survivor group.

Cell-associated ACE has significantly higher catalytic activity as compared to circulating ACE [18]. Patients with severe ARDS may sustain more lung injury to the endothelium and epithelium thus reducing the levels of cell-associated enzymes which corresponded with increased SOFA scores and lactate serum levels. Other evidence supporting decreased enzymatic activity in the non-survivor group may be inferred by comparing peptide ratios (product/reactant) between the two groups. Survivors had higher levels of downstream products as suggested by higher peptide ratios of A(1–10)/A(1–12), A(1–8)/A(1–10), A(1–7)/A(1–9) and A(1–7)/A(1–8). The small sample size may account for why median levels of some peptide ratios such as A(1–7)/A(1–9) and A(1–7)/A(1–8) were not significantly different between survival and non-survival groups. Reduction in ACE and ACE2 enzymatic activity may ultimately lead to accumulation of A(1–10) in patients who succumb to ARDS. Therefore, elevated levels of A(1–10) at study initiation may serve as a useful marker to predict non-survival amongst ARDS patients.

Although non-significant, survivors had higher plasma concentrations of A(1–7) and A(1–9) during the study period that may have driven better tissue regeneration. The importance of the A(1–7)/ACE2 axis has been shown in animal models of lung injury [8, 14]. In these studies, administration of exogenous A(1–7) or ACE2 in ARDS mouse models attenuated the inflammatory response, decreased lung injury and improved oxygenation. ACE2 can also promote the formation of A(1–9) where its binding to AT_2_ can promote tissue regeneration. A role for A(1–9) in attenuating cardiac fibrosis in rats by binding to the AT_2_ receptor was previously shown [18, 19]. AT_2_ is a fetal receptor expressed in response to wound injury, suggesting a role in tissue repair and regeneration. Not only is A(1–9) an active peptide metabolite that can activate AT_2_, it can be further metabolized to A(1–7) via ACE-mediated metabolism. We speculate that one reason non-survivors had declining P/F ratios over the study period and in turn a higher mortality is due to loss of the protective mechanism afforded by ACE2. Reduced ACE2 activities may cause a decrease in A(1–9) and A(1–7) production, which can activate AT_2_ and Mas, respectively. In this study, there were no significant differences in A(1–7) or A(1–9) (Table 2) between the groups. However elevated levels of A(1–10) suggest that there is a need for additional pro-resolving RAS peptides in response to wound healing. A larger study is needed to confirm clinical significance.

We also evaluated whether other factors influenced the levels of RAS peptides. The influence of BMI on outcomes in ARDS is unclear. While some studies show decreased mortality associated with higher BMI, other studies have shown that obesity has no effect [20, 21]. A more recent meta-analysis showed that obesity confers a protective effect and thus a decrease in mortality [22]. In the current study, we did not detect any relationship between obesity and increased mortality. Of note, obesity can result in higher levels of circulating angiotensin levels, but BMI was not statistically different between the survivor and non-survivor groups.

This study also evaluated renal function in relation to mortality. Since kidneys are an important metabolic site of production of RAS precursors and plasma ACE2 activity is reduced in patients with chronic kidney disease [23], we evaluated the impact of renal dysfunction in relation to increase mortality risk. Although we did not observe an association between renal failure status and ARDS survival, there was a significantly higher A(1–10)/A(1–9) ratio (a marker for ACE2 activity) in the group with normal renal function. Reduction in ACE2 may lead to a multitude of effects including impaired lung and kidney repair due to an inability to transform A(1–8) (a pro-inflammatory factor) to A(1–7) (an anti-inflammatory factor). However, studies have shown that circulating levels of ACE2 are very low and some of the effects of ACE2 are local [24]. Therefore, renal injury and loss of ACE2 would likely only limit kidney repair and not affect lung repair.

There are limitations to this study which include the small sample size (n = 39). Nevertheless, this study highlights an association of the RAS pathway in relation to the pathogenesis of ARDS. The levels of RAS peptides, in particular accumulation of A(1–10), suggest that enzymes such as ACE and ACE2 which are required to form active RAS peptides such as A(1–9), A(1–8) and A(1–7) may not be available. This may be a consequence of lung injury where lung parenchymal cell injury and death release ACE and ACE2, significantly lowering enzymatic activity(ies). This may explain the accumulation of A(1–10) at study entry, and serve as a useful predictive marker for disease severity. It would have been ideal to quantify the enzymatic activites to clearly dissect the mechanism(s) leading to clinical outcomes and probe Ang-(1–10) variations in synthesis. However, the presence of protease inhibitors in the blood samples as a requirement for metabolite analysis did not permit us to do such quantification. Larger studies are needed to establish a causative role for the RAS pathway in ARDS, including evaluation of the overall enzymatic activity of ACE and ACE2 in ARDS.

## Conclusion

A(1–10) accumulation and reduced A(1–9) concentration in the non-survivor group suggest that ACE2 activities may be reduced in patients succumbing to ARDS. Plasma levels of both A(1–10) and A(1–9) and their ratio may serve as useful biomarkers for prognosis in ARDS patients.

## References

[1] Park WY, Goodman RB, Steinberg KP, Ruzinski JT, Radella F, Park DR, et al Cytokine balance in the lungs of patients with acute respiratory distress syndrome. American journal of respiratory and critical care medicine. 2001;164(10):1896–903. 10.1164/ajrccm.164.10.2104013. 11734443

[2] Rubenfeld GD, Caldwell E, Peabody E, Weaver J, Martin DP, Neff M, et al Incidence and outcomes of acute lung injury. New England Journal of Medicine. 2005;353(16):1685–93. 10.1056/NEJMoa050333. 16236739

[3] Bellani G, Laffey JG, Pham T, Fan E, Brochard L, Esteban A, et al Epidemiology, patterns of care, and mortality for patients with acute respiratory distress syndrome in intensive care units in 50 countries. Jama. 2016;315(8):788–800. 10.1001/jama.2016.0291. 26903337

[4] Calfee CS, Gallagher D, Abbott J, Thompson BT, Matthay MA, Network NA. Plasma angiopoietin-2 in clinical acute lung injury: prognostic and pathogenetic significance. Critical care medicine. 2012;40(6):1731 10.1097/CCM.0b013e3182451c87. 22610178 PMC3601049

[5] Levitt JE, Gould MK, Ware LB, Matthay MA. Analytic review: the pathogenetic and prognostic value of biologic markers in acute lung injury. Journal of intensive care medicine. 2009;24(3):151–67. 10.1177/0885066609332603. 19282296

[6] Imai Y, Kuba K, Rao S, Huan Y, Guo F, Guan B, et al Angiotensin-converting enzyme 2 protects from severe acute lung failure. Nature. 2005;436(7047):112 10.1038/nature03712. 16001071 PMC7094998

[7] Huang F, Guo J, Zou Z, Liu J, Cao B, Zhang S, et al Angiotensin II plasma levels are linked to disease severity and predict fatal outcomes in H7N9-infected patients. Nature communications. 2014;5:3595 10.1038/ncomms4595. 24800963 PMC7091598

[8] Wösten‐van Asperen RM, Lutter R, Specht PA, Moll GN, van Woensel JB, van der Loos CM, et al Acute respiratory distress syndrome leads to reduced ratio of ACE/ACE2 activities and is prevented by angiotensin‐(1–7) or an angiotensin II receptor antagonist. The Journal of pathology. 2011;225(4):618–27. 10.1002/path.2987. 22009550

[9] Lijnen P, Petrov V, Fagard R. Induction of cardiac fibrosis by angiotensin II. Methods and findings in experimental and clinical pharmacology. 2000;22(10):709–24. 10.1358/mf.2000.22.10.802287. 11346891

[10] Lim D-S, Lutucuta S, Bachireddy P, Youker K, Evans A, Entman M, et al Angiotensin II blockade reverses myocardial fibrosis in a transgenic mouse model of human hypertrophic cardiomyopathy. Circulation. 2001;103(6):789–91. 10.1161/01.CIR.103.6.789. 11171784 PMC2779524

[11] Donoghue M, Hsieh F, Baronas E, Godbout K, Gosselin M, Stagliano N, et al A novel angiotensin-converting enzyme–related carboxypeptidase (ACE2) converts angiotensin I to angiotensin 1–9. Circulation research. 2000;87(5):e1–e9. 10.1161/01.RES.87.5.e1. 10969042

[12] Reddy Gaddam R, Chambers S, Bhatia M. ACE and ACE2 in inflammation: a tale of two enzymes. Inflammation & Allergy-Drug Targets (Formerly Current Drug Targets-Inflammation & Allergy). 2014;13(4):224–34. 10.2174/1871528113666140713164506 25019157

[13] e Silva AS, Silveira K, Ferreira A, Teixeira M. ACE2, angiotensin‐(1‐7) and Mas receptor axis in inflammation and fibrosis. British journal of pharmacology. 2013;169(3):477–92. 10.1111/bph.12159. 23488800 PMC3682698

[14] e Silva ACS, Teixeira MM. ACE inhibition, ACE2 and angiotensin-(1⿿ 7) axis in kidney and cardiac inflammation and fibrosis. Pharmacological research. 2016;107:154–62. 10.1016/j.phrs.2016.03.018. 26995300

[15] Zambelli V, Bellani G, Borsa R, Pozzi F, Grassi A, Scanziani M, et al Angiotensin-(1–7) improves oxygenation, while reducing cellular infiltrate and fibrosis in experimental Acute Respiratory Distress Syndrome. Intensive care medicine experimental. 2015;3(1):8 10.1186/s40635-015-0044-3. PMC4512981 26215809

[16] Force ADT, Ranieri V, Rubenfeld G. Acute respiratory distress syndrome. Jama. 2012;307(23):2526–33. 10.1001/jama.2012.5669 22797452

[17] Vittinghoff E, Glidden DV, Shiboski SC, McCulloch CE. Regression methods in biostatistics: linear, logistic, survival, and repeated measures models: Springer Science & Business Media; 2011.

[18] Flores-Munoz M, Work LM, Douglas K, Denby L, Dominiczak AF, Graham D, et al Angiotensin-(1–9) attenuates cardiac fibrosis in the stroke-prone spontaneously hypertensive rat via the angiotensin type 2 receptor. Hypertension. 2011:HYPERTENSIONAHA. 111.177485. 10.1161/HYPERTENSIONAHA.111.177485 22184331

[19] Jiang F, Yang J, Zhang Y, Dong M, Wang S, Zhang Q, et al Angiotensin-converting enzyme 2 and angiotensin 1–7: novel therapeutic targets. Nature Reviews Cardiology. 2014;11(7):413 10.1038/nrcardio.2014.59. 24776703 PMC7097196

[20] O'Brien JM, Welsh CH, Fish RH, Ancukiewicz M, Kramer AM. Excess body weight is not independently associated with outcome in mechanically ventilated patients with acute lung injury. Annals of internal medicine. 2004;140(5):338–45. 10.7326/0003-4819-140-5-200403020-00009. 14996675

[21] Memtsoudis SG, Bombardieri AM, Ma Y, Walz JM, Chiu YL, Mazumdar M. Mortality of patients with respiratory insufficiency and adult respiratory distress syndrome after surgery: the obesity paradox. Journal of intensive care medicine. 2012;27(5):306–11. 10.1177/0885066611411410. 21778465

[22] Zhi G, Xin W, Ying W, Guohong X, Shuying L. “Obesity paradox” in acute respiratory distress syndrome: asystematic review and meta-analysis. PloS one. 2016;11(9):e0163677 10.1371/journal.pone.0163677. 27684705 PMC5042414

[23] Roberts MA, Velkoska E, Ierino FL, Burrell LM. Angiotensin-converting enzyme 2 activity in patients with chronic kidney disease. Nephrology Dialysis Transplantation. 2013;28(9):2287–94. 10.1093/ndt/gft038. PMC7537611 23535224

[24] Ferrario CM, Jessup J, Chappell MC, Averill DB, Brosnihan KB, Tallant EA, et al Effect of angiotensin-converting enzyme inhibition and angiotensin II receptor blockers on cardiac angiotensin-converting enzyme 2. Circulation. 2005;111(20):2605–10. 10.1161/CIRCULATIONAHA.104.510461. 15897343

